# Excessive Checking in Obsessive-Compulsive Disorder: Neurochemical Correlates Revealed by 7T Magnetic Resonance Spectroscopy

**DOI:** 10.1016/j.bpsgos.2023.08.009

**Published:** 2023-08-25

**Authors:** Marjan Biria, Paula Banca, Engin Keser, Máiréad P. Healy, Stephen J. Sawiak, Ana Maria Frota Lisbôa Pereira de Souza, Aleya A. Marzuki, Akeem Sule, Trevor W. Robbins

**Affiliations:** a Department of Psychology, University of Cambridge, Cambridge, United Kingdom; b Behavioural and Clinical Neuroscience Institute, University of Cambridge, Cambridge, United Kingdom; c Division of Psychiatry and Division of Psychology and Language Sciences, University College London, London, United Kingdom; d Wolfson Brain Imaging Centre, Department of Clinical Neurosciences, University of Cambridge, Cambridge, United Kingdom; e Department of Psychology, School of Medical and Life Sciences, Sunway University, Selangor, Malaysia; f Department of Psychiatry, School of Clinical Medicine, University of Cambridge, Cambridge, United Kingdom

**Keywords:** 7T-MRS, Anterior cingulate cortex, Checking, GABA, Glutamate, OCD

## Abstract

**Background:**

Compulsive checking, a common symptom of obsessive-compulsive disorder (OCD), has been difficult to capture experimentally. Therefore, determination of its neural basis remains challenging despite some evidence suggesting that it is linked to dysfunction of cingulostriatal systems. This study introduces a novel experimental paradigm to measure excessive checking and its neurochemical correlates.

**Methods:**

Thirty-one patients with OCD and 29 healthy volunteers performed a decision-making task requiring them to decide whether 2 perceptually similar visual representations were the same or different under a high-uncertainty condition without feedback. Both groups underwent 7T magnetic resonance spectroscopy scans on the same day. Correlations between out-of-scanner experimental measures of checking and the glutamate/GABA (gamma-aminobutyric acid) ratio in the anterior cingulate cortex, supplementary motor area, and occipital cortex were assessed. Their relationship with subjective ratings of doubt, anxiety, and confidence was also investigated.

**Results:**

Patients with OCD exhibited excessive and dysfunctional checking, which was significantly correlated with changes in the glutamate/GABA ratio within the anterior cingulate cortex. No behavioral/neurochemical relationships were evident for either the supplementary motor area or occipital cortex. The excessive checking observed in patients was negatively correlated with their confidence levels and positively related to doubt, anxiety, and compulsivity traits.

**Conclusions:**

We conclude that experimental measures of excessive and dysfunctional checking in OCD, which have been linked to increased doubt, anxiety, and lack of confidence, are related to an imbalance between excitatory and inhibitory neural activity within the anterior cingulate cortex. This study adds to our understanding of the role of this region in OCD by providing a laboratory model of the possible development of compulsive checking.

Performing functional checking is essential for gathering survival information. However, excessive checking without a clear purpose can be time-consuming, highly debilitating, and stressful. This is typically true of obsessive-compulsive disorder (OCD), in which repeated checking is the most commonly reported symptom (79.3%) (1). Checking, related to the dimensions of “incompleteness” and “disturbing thoughts” (harm/check), was also highlighted as an important feature in a recent analysis of OCD symptoms (2). Several studies have endeavored to measure compulsive checking in the laboratory, but the findings have been mixed. Some studies have found excessive checking in patients with OCD on perceptual decision-making tasks (3,4), while others have not replicated the results (5,6). These inconsistencies are likely due to the studies’ focusing on measuring different cognitive constructs, which may contribute differently to compulsive checking, such as intolerance of uncertainty (7,8), inflated sense of responsibility/harm avoidance (9,10), memory performance (11), metacognition and cognitive confidence (12, 13, 14, 15), or anxiety (16,17).

The insights we have so far on the neural basis of excessive checking have come mainly from functional magnetic resonance imaging studies using symptom provocation paradigms (18, 19, 20, 21). Mataix-Cols *et al.* (18) examined the neural response to pictures associated with checking behavior (e.g., electrical appliances, doors), while others (19,21) investigated differential neural activation during symptom provocation in groups of OCD checkers and washers compared with control participants. These studies suggest that checking is linked to the striatum, thalamus, and anterior cingulate cortex (ACC) (18,19).

We present a novel experimental paradigm that is optimized to measure excessive checking in patients with OCD that also unusually characterizes its functionality in terms of the way in which checking-derived information is deployed to improve performance as the main task goal [see also (22)]. The paradigm taps into uncertainty and perceptual decision making because 1) patients with OCD (23) and nonclinical high-compulsive individuals (24) have been shown to exhibit higher decision thresholds under uncertainty, and 2) checking paradigms involving perceptual decision making have reliably induced checking in OCD (6). The task is perceptually simple yet sufficiently difficult to induce checking. We have also incorporated a measure of confidence to further assess its interrelationships with uncertainty, doubt, and checking.

We also investigated possible neurochemical mechanisms underlying compulsive checking by combining our checking paradigm with measurements of glutamate (Glu) and GABA (gamma-aminobutyric acid) acquired using magnetic resonance spectroscopy (MRS). These neurotransmitters are critical to the functioning of the frontostriatal-thalamic circuits that are involved in the neuropathology of OCD, as evidenced by metabolic studies that have found altered Glu and GABA concentrations in the ACC (25, 26, 27, 28, 29) and prefrontal cortex (30, 31, 32) regions. However, their exact role in the pathophysiology of OCD is unknown given the mixed findings (33,34), which are likely due to the use of lower magnet strengths and thus less specificity (e.g., Glu plus glutamine were measured together as Glx). Here, we used a high-resolution 7T scanner, which enabled reliable isolation of Glu and GABA quantification from glutamine (their precursor). We placed voxels in the ACC and supplementary motor area (SMA) (which included regions from the pre-SMA) given their relevance for checking behavior, as well as in the occipital cortex (OCC), a comparison region. The ACC is crucial for error monitoring (35,36) and reward prediction errors (37,38). The pre-SMA and SMA are regions within the sensorimotor circuit that are known to be relevant for response inhibition and motor compulsions (39) and effective targets for brain stimulation (40, 41, 42, 43). We recently found that Glu and GABA levels were significantly correlated in the 3 regions investigated in healthy volunteers but not in the OCD group, suggesting that a dysfunctional balance may contribute to the pathophysiology of OCD (44). Specifically, patients with OCD had significantly higher levels of Glu and lower levels of GABA in the ACC, resulting in significantly higher Glu/GABA ratios. In the SMA, however, despite there being no absolute group differences in Glu, Glu/GABA levels were related to a habitual index in both patients with OCD and healthy individuals with compulsive traits. No significant group differences were found in Glu or GABA in the OCC (44). In the study presented here, we investigated how this excitatory/inhibitory balance relates to functional and dysfunctional checking within these regions. We hypothesized that patients with OCD would demonstrate excessive and dysfunctional checking (i.e., checking that does not improve performance on the task). We also hypothesized that this behavior would be related to imbalanced Glu/GABA ratios within the ACC and SMA. We expected these effects to be a rather general tendency in OCD, including not only the “checking” subtype, but also “washers” and “orderers” in our mixed sample. Finally, we hypothesized that such aberrant behavior and neurochemical imbalance might be accompanied by higher levels of anxiety, habitual tendencies, or intolerance of uncertainty.

## Methods and Materials

### Participants

Participants included 29 healthy volunteers (HVs) (48% female) and 31 patients with OCD (55% female) who were fluent English speakers and were matched for age, sex, and IQ. The data collection started on October 7, 2020, and ended on June 8, 2022. The demographic and clinical characteristics of both groups are shown in Table 1. Participants were the same as those in our recent study (44) except for the healthy group, from which 1 participant was excluded from the current study due to incomplete behavioral data.

**Table 1:** Clinical and Demographic Information for HVs and Patients With OCD

	OCD (*n* = 31), Mean ± SD	HV (*n* = 29), Mean ± SD	Statistic	*p*	Cohen’s *d* or *η_p_^2^*	95% CI
Age, Years	30.79 ± 9.99	32.48 ± 13.00	*t*_58_ = −0.56	.57	*d* = −0.14	−7.69 to 4.29
Sex, Female	55%	48%	χ^2^_1_ = 0.25	.61	*d* = −0.06 (Phi)	0.034 to 0.097
Education, Years	15.35 ± 2.85	17.31 ± 2.73	*t*_58_ = −2.7	.009	*d* = −0.69	−3.40 to −0.50
Verbal IQ	110.21 ± 6.13	113.15 ± 7.89	*t*_58_ = −1.62	.11	*d* = −0.41	−6.58 to 0.69
MADRS	17.65 ± 11.01	5.24 ± 3.72	*t*_37.2_ = 5.92	<.001	*d* = 1.48	8.15 to 16.64
STAI-State	37.65 ± 9.12	27.96 ± 6.79	*t*_58_ = 4.63	<.001	*d* = 1.19	5.50 to 13.85
STAI-Trait	56.55 ± 11.03	37.72 ± 10.49	*t*_58_ = 6.76	<.001	*d* = 1.74	13.25 to 24.39
YBOCS Total	22.94 ± 5.90	NA	NA	NA	NA	NA
YBOCS Obsessions	11.45 ± 3.21	NA	NA	NA	NA	NA
YBOCS Compulsions	11.52 ± 2.97	NA	NA	NA	NA	NA
OCI Total	63.32 ± 30.37	6.38 ± 5.78	*U* = 893	<.001	η_p_^2^ = 0.74	45.00 to 66.00
OCI Washing	10.48 ± 9.86	1.03 ± 1.40	*U* = 764	<.001	η_p_^2^ = 0.38	2.00 to 11.00
OCI Checking	13.06 ± 8.28	1.27 ± 1.46	*U* = 837	<.001	η_p_^2^ = 0.57	9.00 to 14.00
OCI Doubting	6.26 ± 3.56	0.24 ± 0.63	*U* = 844	<.001	η_p_^2^ = 0.64	4.00 to 9.00
OCI Ordering	7.29 ± 5.24	1.34 ± 1.49	*U* = 806	<.001	η_p_^2^ = 0.49	3.00 to 7.00
OCI Obsessions	14.42 ± 8.11	0.76 ± 1.21	*U* = 865	<.001	η_p_^2^ = 0.67	11.00 to 16.00
OCI Hoarding	3.16 ± 2.86	0.96 ± 1.74	*U* = 669	<.001	η_p_^2^ = 0.19	1.00 to 3.00
OCI Neutralization	8.65 ± 5.77	0.76 ± 0.95	*U* = 847	<.001	η_p_^2^ = 0.61	4.00 to 10.00
IUS	84.61 ± 22.85	54.38 ± 17.23	*t*_58_ = 5.75	<.001	*d* = 1.48	19.71 to 40.75
HTQ Compulsivity	23.87 ± 4.21	13.82 ± 5.51	*t*_58_ = 7.96	<.001	*d* = 2.05	7.51 to 12.56

For the healthy group, 1 participant did not provide OCI data (*n* = 28 for HV OCI data). For the OCD group, all data were complete. Two-sided tests were used for age, sex, education, and verbal IQ. The rest of the variables were tested using a one-sided test. χ^2^ test is used for categorical data; Phi is a measure of effect size for the χ^2^ test; η_p_^2^ is a measure of effect size for the *U* test; and *U* refers to Mann-Whitney *U* test for non-normally distributed data.

HTQ, Habitual Tendencies Questionnaire; HV, healthy volunteer; IUS, Intolerance of Uncertainty Scale; MADRS, Montgomery–Åsberg Depression Rating Scale; NA, not applicable; OCD, obsessive-compulsive disorder; OCI, Obsessive-Compulsive Inventory; STAI, State-Trait Anxiety Inventory; YBOCS, Yale-Brown Obsessive Compulsive Scale.

Healthy participants were recruited from the community, were in good health, had no history of neurological or psychiatric conditions, and were unmedicated. Patients with OCD were recruited through an approved advertisement on the OCD action website (http://www.ocdaction.org.uk) and local support groups and via clinicians in East Anglia. All patients were screened by a qualified psychiatrist using the Mini-International Neuropsychiatric Interview (45) to confirm the OCD diagnosis (as per the DSM-5 criteria) and the absence of comorbid psychiatric conditions. OCD symptom severity and characteristics were measured using the Yale-Brown Obsessive Compulsive Scale (46) within the patient group. For both groups, mood status was assessed using the Montgomery–Åsberg Depression Rating Scale (47), anxiety levels were evaluated using the State-Trait Anxiety Inventory (STAI) (48), and verbal IQ was measured using the National Adult Reading Test (49). We only included patients with OCD who had Yale-Brown Obsessive Compulsive Scale scores higher than 12 (50) and anxiety and depressive symptoms that were directly related to their primary OCD diagnosis. Six patients were unmedicated, and of the 25 medicated patients, 24 were treated with selective serotonin reuptake inhibitors, of whom one was also on a beta-blocker and olanzapine, and a single patient was on the serotonergic tricyclic drug clomipramine and a beta blocker. General exclusion criteria for both groups were substance dependence, neurological or medical illnesses, and head injury. All participants had normal or corrected-to-normal vision and hearing and completed the following self-report questionnaires: The Obsessive-Compulsive Inventory (OCI) (51), a self-report measure of obsessive-compulsive symptoms; the Intolerance of Uncertainty Scale (52), a self-report measure of uncertainty unpleasantness; and the compulsivity subscale of the Habitual Tendencies Questionnaire (53).

A mixed group of patients with OCD was recruited for this study; patients were not specifically selected based on their symptom categories (e.g., OCD checkers or washers). Of the 31 patients who were assessed, 7 showed predominant checking symptoms, and 3 patients had predominantly washing compulsions based on their OCI checking and washing subscale scores. The remainder presented a mixture of compulsive behaviors such as washing, checking, and ordering. OCI subscale scores for washing, checking, and ordering for all participants are shown in Table S1.

This study was approved by the East of England—Cambridge South Research Ethics Committee (16/EE/0465). Written informed consent was collected from all participants before they began the testing session, and all participants received monetary compensation for their participation.

### Image Verification Task

**Figure 1:**
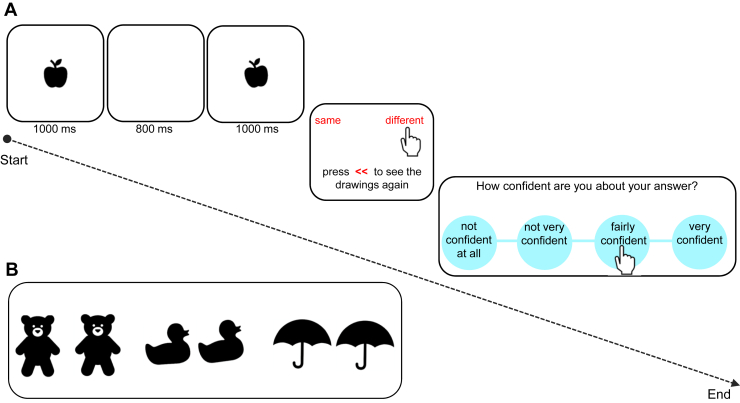
Image verification task. **(A)** An example of a trial, with 2 apples differing in angle, displayed for 1 second, with a white interstimulus interval presented for 800 ms in between. Participants could decide immediately whether the 2 images were the same or different by pressing the words on the screen or they could press the red << sign to go back and check the images again. Once a decision had been made, participants were asked to rate how confident they were about their answers using a 4-point scale as depicted in the third panel. **(B)** Three examples of black-and-white stimuli used in this task. From left to right: 2 bears (the right bear is bigger), ducks (the right duck is more crooked), and umbrellas (the 2 umbrellas are exactly the same).

The image verification task (IVT) measures perceptual decision making and checking. On each trial, participants were required to inspect 2 images showing black-and-white drawings of objects displayed on a computer screen one after another and decide whether the 2 images were the same or different (Figure 1). They were instructed to be as accurate as possible. Before they gave their answers, participants were given the chance to observe the images again as many times as they liked. The total number of times participants chose this option to view the images again was used as a measure of checking. All participants were explicitly told, “you can go back and check the images again to decide if they are the same or different.” Once a decision had been made, participants then rated how confident they were about their answer on a 4-point ranging from “not confident at all” to “very confident,” with “not very confident” and “fairly confident” choices in between. The task comprised 45 trials: 5 practice trials and 40 experimental trials, which were delivered on a 13-inch touchscreen Dell laptop computer.

This conceptually simple task was nonetheless sufficiently perceptually difficult to induce uncertainty. High uncertainty was achieved by providing no feedback on performance throughout the task and by changing the size or the angle of the images very slightly (either 0% or 5% change), which made it difficult to decide whether the 2 images were exactly the same or slightly different. There were 5 practice trials, of which 3 were easy trials, i.e., with clear differences in size, angle (20% change), or shape (e.g., 2 different car types). Only 1 attribute (either size, angle, or shape) changed on each trial (not more than 1 aspect at a time). Each 1-second image presentation was separated by an interstimulus white screen (800 ms). Main measures were accuracy of choices, checking rates, and confidence ratings. The task was performed outside the scanner immediately before or after completing the scan.

### MRS Data Acquisition

**Figure 2:**
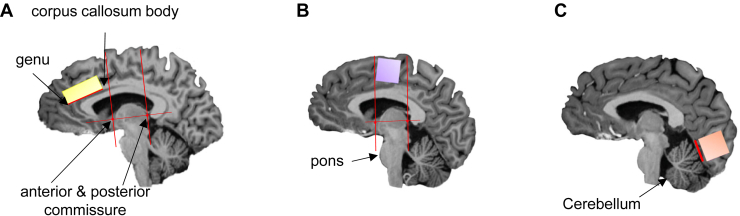
Voxel positions and landmarks. The location of the 3 voxels is shown in a sagittal space while indicating the landmarks that were used to increase their consistent placement and to avoid an overlap between the anterior cingulate cortex and supplementary motor area (SMA) voxels. First, a horizontal line was drawn between the anterior and posterior commissure with 2 vertical lines going through each of them perpendicularly. These lines are depicted in red for the anterior cingulate cortex and SMA voxels. This figure is reproduced from Figure S1 in Biria *et al.* (44). **(A)** The anterior cingulate cortex box (in yellow) was placed in front of the line going through the anterior commissure, with the outer left corner of the box being in front of the genu of the corpus callosum. **(B)** The SMA box (in purple) was placed above the pons and between the 2 red lines. The upper side of the box was placed parallel with the skull above it. **(C)** Finally, the occipital cortex box (in orange) was placed in the outermost corner of the occipital lobes while avoiding the skull and sinuses, with the lower side of the box being parallel with the red line above the cerebellum. All 3 voxels were placed bilaterally, and the SMA box included regions from the SMA and the pre-SMA.

The proton MRS (^1^H-MRS) scans took place at the Wolfson Brain Imaging Centre, University of Cambridge (United Kingdom). Whole-brain T1-weighted MR and single-voxel proton MRS scans were acquired using a 7T Terra magnetic resonance imaging scanner (Siemens). The scanner was equipped with a Nova single-channel transmit and a 32-channel array head coil for signal reception (Nova Medical). T1-weighted magnetization-prepared 2 rapid acquisition gradient-echo (54) images were acquired to aid with positioning of voxels and used in the analysis to perform within-subject tissue corrections. See Biria *et al.* (44), a study in which the same sample of subjects and procedure were used, for details about the MRS data acquisition, preparation, and analysis. Clear landmarks were used to place 3 voxels bilaterally: at the ACC (12 × 20 × 33 mm^3^), at the SMA, and at the OCC (the latter 2 were square-shaped voxels [20 × 20 × 20 mm^3^]). All voxels (each of which lasted for about 20 minutes) were placed bilaterally in the midline so that measurements from both hemispheres could be acquired simultaneously. This helped to increase the amount of gray matter within each box, leading to a higher signal-to-noise ratio. All the voxels were located manually by the same researcher (MB) to increase the reliability of the voxel placements across participants (see Figure 2 for voxel positions and landmarks). Table S2 shows the MRS checklist according to a recent consensus by Lin *et al.* (55).

Segmentation analysis, including partial volume corrections within participants, was performed using SPM12. The magnetization-prepared 2 rapid acquisition gradient-echo images were used to extract tissue fractions for each participant (gray matter, white matter, and cerebrospinal fluid). Following the recommendation of Kreis *et al.* (56), a straight cutoff score for Cramér-Rao lower bound was avoided to prevent exclusion of extreme values that may be disorder/group specific. Instead, according to Frangou *et al.* (57), values larger than 2 SD from each group mean were excluded per individual metabolites using both Cramér-Rao lower bound and metabolite concentration values relative to Cr+PCr (creatine plus phosphocreatine). The following data were excluded: within the SMA voxel, GABA in 1 HV and 1 participant with OCD and glutamine in 2 patients with OCD; within the OCC voxel, Glu and glutamine in 2 HVs, GABA in 1 HV, and GABA in 3 patients with OCD. One ACC and 1 OCC voxel were excluded for 1 patient with OCD due to an error during data collection. LCModel (58) version 6.2-3 was used with an automated fitting routine to quantify the relevant metabolites.

### Statistical Analysis

For group comparison, an independent samples *t* test was used on descriptive data, clinical measures, and task performance outcomes. When the normality condition was not satisfied, a Mann-Whitney *U* test was used. For correlational analysis, when the data were not normally distributed, the Spearman rank *r*_s_ correlation coefficient was used instead of Pearson’s *r*. The average checking rates, accuracy of answers, and confidence ratings were used as dependent variables and compared between groups. Next, the relationships between task primary outcome measures and the glutamate/GABA ratio in the ACC, SMA, and OCC (the latter being a comparison region) as well as their relationship with clinical symptoms were compared between groups. Two patients with OCD who were outliers for checking (>2 SDs from the mean) were excluded from the correlational analysis of clinical data because they were outliers for clinical scores as well. We reported *p* values <.05 corrected for false discovery rate (FDR), according to the Benjamini-Hochberg method (59).

These corrections were made for 1) behavioral correlations between checking, accuracy, and confidence (4 comparisons); 2) correlations between checking and clinical scales (8 comparisons); and 3) correlations between Glu/GABA levels and checking and accuracy in both groups and per individual voxel (4 comparisons per voxel). The *p* values before the correction are mentioned in the Supplement. The source code used for the FDR calculations can be found here: https://github.com/carbocation/falsediscovery. Python version 3.7.6 was used to perform data analysis.

## Results

Compared with HVs, patients with OCD scored higher on all clinical measures and self-report questionnaires: obsessive-compulsive symptoms as assessed by the OCI, including the checking subscale; compulsivity as assessed by the Habitual Tendencies Questionnaire; depression as assessed by the Montgomery–Åsberg Depression Rating Scale; state and trait anxiety (STAI-S and -T), and intolerance of uncertainty as assessed on the Intolerance of Uncertainty Scale (*p* < .001).

### Behavioral Results

**Figure 3:**
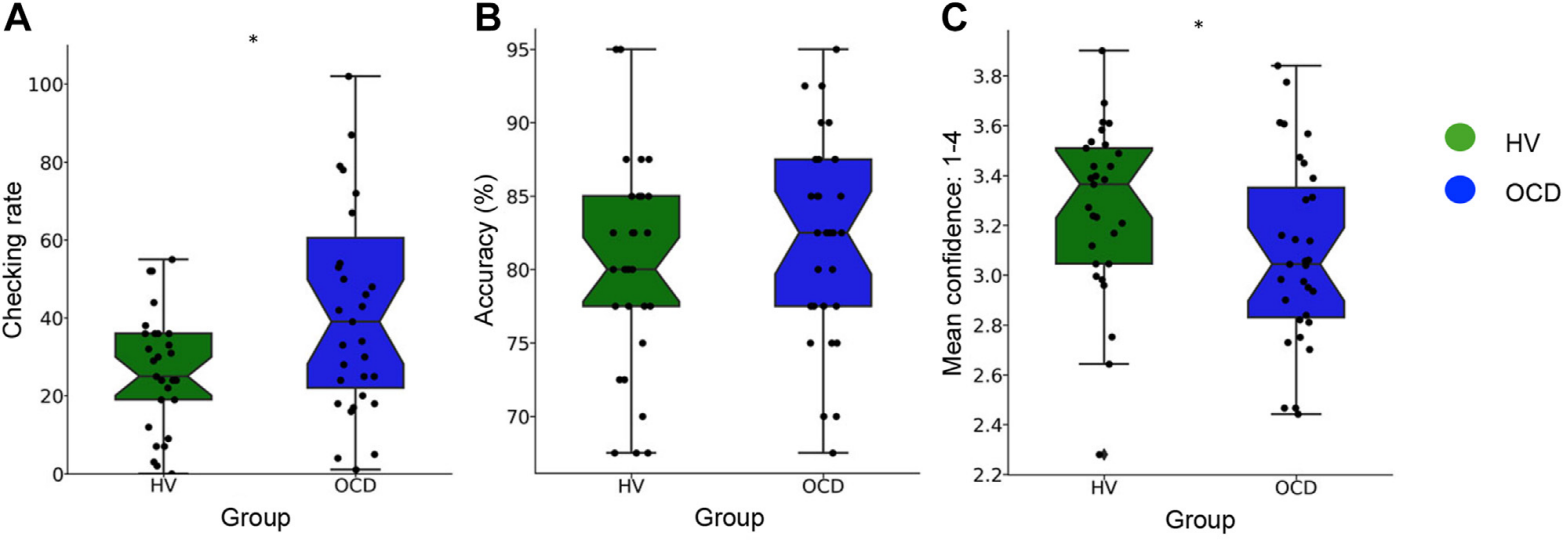
Image verification task results. Boxplots for healthy volunteers (HVs) (*N* = 29) in green and patients with obsessive-compulsive disorder (OCD) (*N* = 31) in blue on measures of **(A)** checking rate, **(B)** accuracy of choices (%), and **(C)** mean confidence ratings (ranging from 1 to 4). For visualization purposes, 2 patients who checked more than 2 SDs from the mean are excluded from these figures; however, they are still included in the analysis. The black circles show the individual data points; the boxes start from the first to the third quartile, with a horizontal line and a notch through the median. The whiskers go from each quartile to the minimum and maximum. The notch approximates a 95% CI for the median. If the notches of 2 boxes do not overlap, this suggests that the medians are significantly different. The points outside whiskers represent the outliers. ∗*p* < .05.

Figure 3 shows the behavioral performance on the IVT for both groups. Despite similar levels of accuracy (*t*_58_ = −0.96, *p* = .34), patients with OCD checked significantly more often than healthy individuals (*U* = 597, *p* = .01) and were less confident in their decisions (*t*_58_ = −1.95, *p* = .05). In addition, patients with OCD took significantly longer to complete the task (HVs: mean = 8.29 minutes, SD = 7.66, patients with OCD: mean = 12.64 minutes, SD = 12.10; *U* = 618, *p* = .006). Descriptive statistics were as follows: accuracy (%) (HVs: mean = 80.25, SD = 7.26, patients with OCD: mean = 82.00, SD = 6.90), checking rate (HVs: mean = 26.24, SD = 15.01, patients with OCD: mean = 48.19, SD = 40.00), and average confidence (HVs: mean = 3.27, SD = 0.34, patients with OCD: mean = 3.08, SD = 0.37).

**Figure 4:**
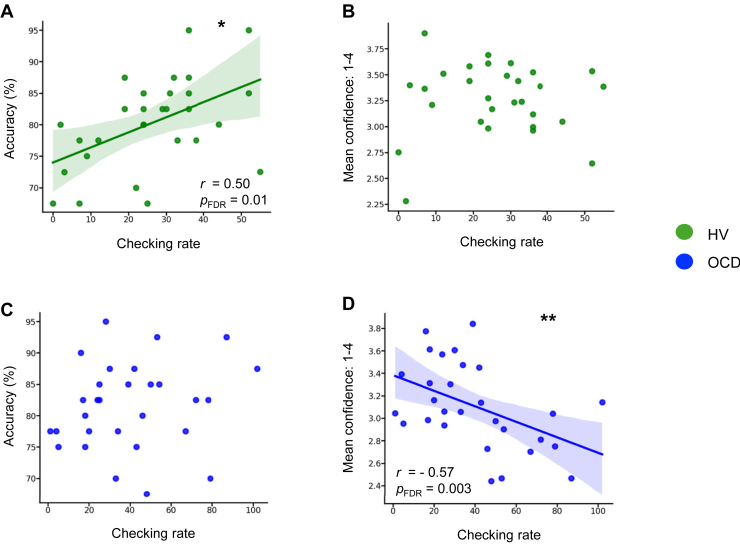
Checking rate relationships with accuracy and confidence. This figure depicts the checking rate relationship with both accuracy (left) and confidence ratings (right). Panels **(A)** and **(B)** show these relationships for healthy volunteers (HVs) (*N* = 29) in green, whereas panels **(C)** and **(D)** show the relationships for patients with obsessive-compulsive disorder (OCD) (*N* = 31) in blue. The fitted lines are drawn only for significant relationships. For visualization purposes, 2 patients who checked more than 2 SDs from the mean are excluded from these figures but not from the analysis. The line of best fit is shown with the 95% CIs for the regression estimate in translucent bands around the regression lines. *r* indicates the Pearson correlation coefficient; *r*_s_ indicates to the Spearman rank correlation coefficient, ∗*p* < .05, ∗∗*p* < .005. FDR, false discovery rate.

Because no feedback on performance was provided to participants, checking could be initially used to improve the accuracy of their choices (thus termed functional checking). Therefore, we assessed the relationship between checking and accuracy to investigate the nature (“functional” vs. “dysfunctional”) of the checking responses. Only HVs showed a significant relationship between checking and accuracy (HVs: *r* = 0.50, *p* = .006, *p*_FDR_ = .01; patients with OCD: *r*_s_ = 0.29, *p* = .10, *p*_FDR_ = .13), i.e., exhibited ”functional” checking (Figure 4A). Regarding the relationship between checking and confidence (the latter was assessed irrespective of correct or wrong choices), only patients with OCD showed a significant negative correlation (HVs: *r* = 0.02, *p* = .88, *p*_FDR_ = .88; patients with OCD: *r*_s_ = −0.57, *p* = .0008, *p*_FDR_ = .003) (i.e., more checking was associated with less confidence, Figure 4D).

### Checking: Relationship With Clinical Measures

The checking rate on the IVT was positively correlated with several clinical measures, but only in patients with OCD. These measures were the OCI doubting subscale (patients with OCD: *r* = 0.48, *p* = .008, *p*_FDR_ = .02; HVs: *r* = −0.35, *p* = .06, *p*_FDR_ = .11), the Habitual Tendencies Questionnaire compulsivity factor, a measure of habitual tendency (patients with OCD: *r*_*s*_ = 0.50, *p* = .005, *p*_FDR_ = .02; HVs: *r* = −0.14, *p* = .48, *p*_FDR_ = .64), and trait anxiety as measured with STAI-T (patients with OCD: *r* = 0.47, *p* = .01, *p*_FDR_ = .02; HVs: *r* = −0.33, *p* = .07, *p*_FDR_ = .11); patients with higher anxiety, doubting, and compulsive tendencies generally checked more often. However, the Intolerance of Uncertainty and the Yale-Brown Obsessive Compulsive Scale compulsion ratings were not correlated with patients’ checking rates (*p*s = .58 and .97, *p*_FDR_ = .66 and .97, respectively). When comparing 2 subgroups of patients, those for whom checking symptoms were predominant versus the others (after excluding the 2 outliers for checking rate of >2 SD), they did not differ on the rate of checking on the IVT (*U* = 84, *p* = .37; predominant OCI checking: mean = 40.00, SD = 20.11, mixed-group: mean = 39.90, SD = 28.00).

### Checking: Relationship With Neurometabolites

**Figure 5:**
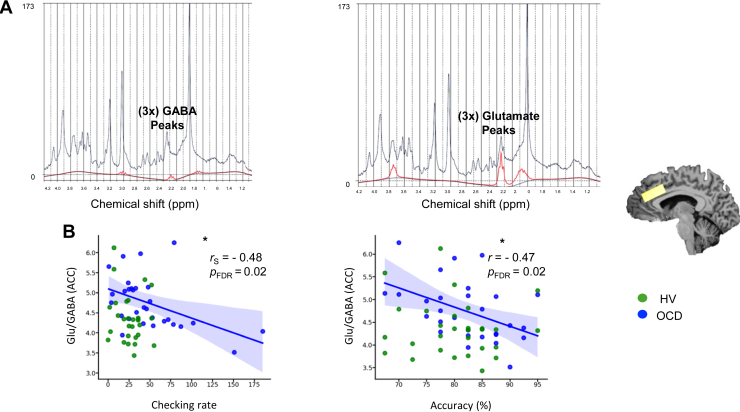
Relationship between image verification task checking rates and brain neurochemical measurements. **(A)** Examples of the LCModel analysis of in vivo ^1^H magnetic resonance spectra acquired from a healthy volunteer (HV) at 7T (semi-LASER, echo time/repetition time = 1.99/4300 ms, from a 20 × 20 × 20 mm^3^ voxel placed bilaterally at the anterior cingulate cortex [ACC]). The acquired spectrum is plotted in black, and the fit is presented in red for GABA (gamma-aminobutyric acid) (left) and glutamate (Glu) (right). **(B)** The correlations between the levels of Glu/GABA concentrations in the ACC and checking rates (left) and accuracy (right). The blue color represents patients with obsessive-compulsive disorder (OCD) (*N* = 30), whereas green depicts the data for HVs (*N* = 29). The fitted lines are drawn only for significant relationships in the OCD group, with the 95% CIs for the regression estimate in translucent bands around the regression lines. The neurometabolites were normalized using (Cr+PCr), corrected for gray and white matter and cerebrospinal fluid of each individual voxel, within participants. *r* indicates Pearson correlation coefficient, and *r*_S_ indicates Spearman rank correlation coefficient. ∗*p* < .05. FDR, false discovery rate; ppm, parts per million.

We further investigated the relationship between the IVT checking rates and the acquired brain neurochemicals to investigate the neural basis of checking. The Glu/GABA levels in the ACC were significantly negatively correlated with both checking and accuracy outcomes of the IVT, but again only in patients with OCD (Figure 5). Statistics are as follows: correlation between Glu/GABA in the ACC and checking rate: (patients with OCD: *r*_*s*_ = −0.48, *p* = .007, *p*_FDR_ = .02; HVs: *r* = −0.17, *p* = .36, *p*_FDR_ = .48); Glu/GABA in the ACC and accuracy correlation: (patients with OCD: *r* = −0.45, *p* = .01, *p*_FDR_ = .02; HVs: *r* = −0.09, *p* = .62, *p*_FDR_ = .62). The latter findings were not due to volume changes within voxels or data “noise” because no differences between groups were found in gray matter, white matter, cerebrospinal fluid, Cramér-Rao lower bound, or signal-to-noise ratio (Table S3). No relationships were found between Glu/GABA levels in the SMA and checking (OCD: *r* = −0.09, *p* = .61, *p*_FDR_ = .73; HVs: *r* = −0.08, *p* = .67, *p*_FDR_ = .73) or accuracy of choices (OCD: *r* = −0.10, *p* = .57, *p*_FDR_ = .73; HVs: *r* = −0.06, *p* = .73, *p*_FDR_ = .73). Similar to the SMA results, no such relationships between OCC Glu/GABA levels and checking (OCD: *r* = −0.21, *p* = .29, *p*_FDR_ = .37; HVs: *r* = −0.24, *p* = .22, *p*_FDR_ = .37) or accuracy (OCD: *r* = −0.18, *p* = .36, *p*_FDR_ = .37; HVs: *r* = 0.19, *p* = .34, *p*_FDR_ = .37) were found for either group.

## Discussion

In this study, individuals with OCD showed significantly elevated levels of dysfunctional checking behavior as measured by our novel perceptual decision-making paradigm. We observed that measures of accuracy and excessive checking in OCD were both negatively correlated with changes in the Glu/GABA ratio within the ACC. We also found that checking was negatively correlated with patients’ confidence levels and positively correlated with doubt (OCI doubt subscale), anxiety (STAI), and a factor on the Habitual Tendencies Questionnaire.

A novel aspect of our laboratory checking task was the assessment of the efficacy or functionality of the checking behavior. Using an explicit instruction about the opportunity to check, the task was sufficiently sensitive to index not only greater levels of checking in the OCD group, but also its lack of significant relationship to accuracy of decision making, confirming that the checking was not always functional in nature. The demonstration of both these effects in a mixed group of patients with OCD, including not only the “checking” subtype but also “washers” and “orderers,” suggests that checking is a rather general tendency in OCD, as reflected in its relative predominance (1). This increased checking tendency was likely determined by a combination of factors such as self-reported lack of confidence and doubt, as well as trait anxiety, especially because anxiety is a predominant symptom in many patients with OCD. We also found a relationship with habitual tendency as measured by the relatively novel Habitual Tendencies Questionnaire scale (53). In fact, this relationship was specific to 1 factor of this scale termed “compulsivity” rather than “routine” or “aversion to novelty” factors. This “compulsivity” factor is based on 4 items that reflect repetitive or perseverative thinking or action, suggesting that the excessive checking behavior has some habitual quality, which is relevant to the hypothesis that patients with OCD may exhibit a bias toward habitual versus goal-directed control over behavior (60,61).

Our findings are consistent with a recent meta-analysis that concluded that laboratory checking paradigms that involve perceptual decision making tend to elicit checking in OCD more consistently (6). Using matching-to-sample tasks analogous to ours, 2 studies (3,4) found that patients with OCD exhibited more checking than healthy control participants, although a third study failed to replicate this result (5). Excessive checking was also found with 3 other tasks (8,9,62). However, these studies, unlike our procedure, did not explicitly assess the functionality or utility of the checking behavior in OCD, which is an important criterion of its compulsive quality. The meta-analysis also hypothesized that task valence (i.e., the emotional tone of the task [reward vs. punishment/threat]) was another possible factor of importance but found the contrary, although the checking paradigm described by Morein-Zamir *et al.* (8) did find more checking in individuals with OCD than in healthy control participants in an appetitive rather than aversive version of the task. We did not use explicit rewarding or punishing feedback for the IVT, and therefore, we assume that the task valence was relatively neutral but nevertheless sufficient to elicit excessive checking in patients with OCD. Despite previous suggestions of sensory impairment in OCD (63,64), we did not find any clear evidence of basic visual sensory deficits in OCD that would lead to lack of confidence in decision making, accuracy on the task being unimpaired.

We also obtained indices of neural function in relation to our behavioral findings in terms of MRS measures at 7T of neurometabolites in the ACC, SMA, and OCC derived from our previous study of the same participants (44). We only observed significant correlations of IVT checking and decision accuracy in the ACC and only in patients with OCD. There seem to be only 3 other neuroimaging studies relevant to checking, although they used the functional magnetic resonance imaging as distinct from the MRS modality and symptom provocation manipulations. Mataix-Cols *et al.* (18) found that OCD checkers had enhanced blood oxygen level–dependent responses in the right ACC (Brodmann area [BA] 32) among a network of other structures including the globus pallidus, the putamen, and various prefrontal (BA 8/9, BA 44) as well as visual (BA 7 and BA 19) regions. Murayama *et al.* (19) found a positive correlation between activated brain areas and symptom severity in the left ACC during symptom provocation, leading them to suggest that the caudate and ACC are associated with checking rituals. In another study (21), checkers demonstrated greater provocation-related activity in the dorsal and medial posterior cingulate cortex and stronger connectivity between the posterior cingulate gyrus and motor cortices, cerebellum, and the right anterior insula/orbitofrontal cortex in response to emotional provocation compared with OCD washers and healthy volunteers. Our findings are more consistent with the former 2 studies in implicating the ACC, although it is likely that this is a node within a more extended neural network that includes the striatum.

Our findings are consistent with theoretical notions of ACC function being implicated in decision making under uncertainty, possibly as part of a cognitive control circuitry that resolves uncertainty and doubt (37,65). This function is likely to be recruited in patients with OCD given their propensity to doubt and lack of confidence and perhaps anxiety, as was also demonstrated by the current findings. It is consistent with evidence of enhanced prediction errors (37,38) and error-related potentials (35,36) in OCD associated with the ACC. Consistent with the behavioral findings of normal accuracy on the IVT, we saw no relationship with neurometabolites in the OCC voxel.

In contrast, IVT accuracy in patients with OCD, while no different from control participants, was negatively correlated with the ACC Glu/GABA ratio (i.e., the higher the ratio, the worse the accuracy), which indicates that this measure of elevated excitatory/inhibitory balance is potentially indicative of a pathophysiological outcome. However, we saw no such relationships in HVs, perhaps because of the relatively easy nature of the task and the lack of evidence of hesitation or anxiety in that group. The apparent recruitment of the ACC in patients with OCD but not in HVs is linked to excessive checking in the IVT generated by their lack of confidence. Therefore, this may reflect a compensatory role of the ACC in decision making as an attempt to resolve conflict and optimize task performance. Although patients who checked most often had the highest accuracy scores (and hence the lowest Glu/GABA ratios), their checking rates were nonetheless excessive. In contrast, patients with worse IVT performance (and hence higher Glu/GABA ratios) tended to check less often, and overall checking was less functional in patients with OCD than in HVs. These relationships were consistent with the negative relationship between checking and Glu/GABA ratio. According to this interpretation, the excessive checking that we observed may indicate what occurs early in the course of a developing compulsion. We hypothesize that, consistent with its lack of significant relationship with decision accuracy, the checking behavior becomes increasingly less goal directed and more habitual and therefore perhaps under greater striatal control [c.f. (18,66)].

Limitations of our study include the medication status of the patients with OCD, although we assume that this cannot account for the significant elevation in dysfunctional checking behavior that we observed. Sample size is also a consideration, although we believe that our study was adequately powered, especially given the improved sensitivity of 7T ^1^H-MRS. We rigorously controlled for multiple correlations using FDR. As recommended for clinical studies of MRS, we included extreme values only when they were within 2 SDs of the group average. Finally, we could not demonstrate that the “checker” subtype in this study (*n* = 7 of 31) showed the greatest levels of checking in the IVT. However, it is unlikely that any particular laboratory checking paradigm would necessarily capture idiosyncratic checking rituals; a patient could have an enhanced checking propensity that is distributed across several typical behaviors and occurs together with other, nonchecking OCD symptoms. However, the implication of the ACC in both laboratory checking and the clinical subtyping of checking (18) suggests that the IVT is indeed relevant to real-life OCD checking.

In conclusion, low confidence and anxiety are factors that contribute to excessive checking in OCD and may determine the degree to which the ACC is recruited as indicated by the Glu/GABA ratio. Given their lack of confidence, patients with OCD may perceive more conflict during task performance than healthy control participants and thus have a greater need to recruit the ACC to resolve such conflict.

## Supplementary Material

Supplementary Data

Key Resources Table
