# Supplementary material for: Excessive Checking in Obsessive-Compulsive Disorder: Neurochemical Correlates Revealed by 7T Magnetic Resonance Spectroscopy
Source: Biol Psychiatry Glob Open Sci. 2023 Aug 25;4(1):363–73. doi: 10.1016/j.bpsgos.2023.08.009 (PMC10829650; doi:10.1016/j.bpsgos.2023.08.009)
Supplement: Key Resources Table [file mmc2.pdf]

SUPPLEMENTARY INFORMATION

Excessive Checking in Obsessive-Compulsive Disorder: Neurochemical Correlates Revealed by 7T Magnetic Resonance Spectroscopy

Biria *et al.*

Table S1 Shows the compulsive symptoms for all subjects measured by the OCI subscales washing, checking and ordering

HV				OCD			
Subjects	Washing	Checking	Ordering	Subjects	Washing	Checking	Ordering
1	3	4	3	1	10	13	7
2	3	4	1	2	1	9	2
3	1	1	1	3	21	23	6
4	0	0	0	4	11	10	4
5	1	4	1	5	0	1	0
6	1	0	0	6	24	3	7
7	0	0	0	7	3	13	10
8	0	0	3	8	21	1	3
9	1	1	1	9	30	13	6
10	0	2	2	10	3	14	5
11	0	0	0	11	7	13	15
12	0	3	1	12	6	1	1
13	0	1	0	13	22	26	7
14	2	1	1	14	1	22	4
15	0	0	1	15	11	15	4
16	0	0	1	16	12	5	4
17	2	1	0	17	21	4	8
18	0	0	0	18	1	1	1
19	1	3	4	19	0	12	7
20	0	0	0	20	2	12	3
21	3	3	3	21	26	13	11
22	2	1	3	22	6	29	6
23	0	0	2	23	23	19	13
24	1	0	0	24	30	26	20
25	4	4	1	25	2	19	19
26	0	0	0	26	0	4	1
27	0	2	1	27	1	14	10
28	0	1	6	28	14	29	10
29	5	1	3	29	6	10	16
				30	2	16	4
				31	8	15	12

Acronyms: HV = healthy volunteers, OCD = obsessive-compulsive disorder, Subj = subject number.

Table S2 MRS checklist according to Lin et al., 2021 (1) and reproduced from Biria et al., 2023 (2).

**Site name:**	Wolfson Brain Imaging Centre, Department of Clinical Neurosciences, University of Cambridge
**1. Hardware**	
**a**. Field strength [T]:	7T
**b.** Manufacturer:	Siemens
**c.** Model (software version if available)	Magnetom 7T Terra
**d.** RF coils: nuclei (transmit/receive), number of channels, type, body part	Nova 1Tx32Rx 1H head coil
**e.** Additional hardware (eg shim inserts, dielectric pads)	None
**2. Acquisition**	
**a.** Pulse sequence	semi-LASER
**b.** Volume of Interest (VOI) locations	bilaterally at anterior cingulate cortex (ACC), supplementary (including pre-supplementary) motor area (SMA), occipital cortex (OCC), see Fig.S1 above.
**c.** Nominal VOI size [cm^3, mm^3]	ACC: 12 x 20 x 33 cm3, SMA: 2 x 2 x 2 cm3, OCC: 2 x 2 x 2 cm3
**d.** Repetition Time (TR), Echo Time (TE)	TR = 5000, total TE = 26ms, TE 1, 2, 3 = 7, 10, 9ms
**e.** Total number of excitations or acquisitions per spectrum in time series for kinetic studies<br>i. Number of Averaged spectra per time-point<br>ii. Averaging method (e.g. block-wise or moving average)<br>iii. Total number of spectra (acquired / in time-series)	64 averages with water-suppression plus spectra without water suppression, and 8 water peak files were collected (4 at the beginning and 4 at the end).
**f.** Additional sequence parameters (spectral width in Hz, number of spectral points, frequency offsets)	The standard CMRR short-TE sLASER protocol were followed.
**g.** Water Suppression Method	VAPOR
**h.** Shimming Method, reference peak, and thresholds for "acceptance of shim" chosen	FASTESTMAP, acceptable shims have linewidth <= 15Hz otherwise manual shimming was performed.
**i.** Triggering or motion correction method (respiratory, peripheral, cardiac triggering, incl. device used and delays)	None
**3. Data analysis methods and outputs**	
**a.** Analysis software	LCModel version 6.2-3, MRspa (Dinesh Deelchand, University of Minnesota, www.cmrr.umn.edu/downloads/mrspa)
**b.** Processing steps deviating from quoted reference or product	No deviations took place in the pre-processing steps from Provencher (2021). At the level of the data analysis, a segmentation analysis was performed using SPM12 and the MP2RAGE images to extract tissue fractions for each subject and each individual voxel for grey matter, white matter and cerebrospinal fluid. Next, partial volume corrections were performed within subjects/voxels according to Harris et al., (2015) for GABA, and Provencher (2021) for the rest of the metabolites.
**c.** Output measure (e.g. absolute concentration, institutional units, ratio)	Ratio to total creatine (creatine + phosphocreatine).
**d.** Quantification references and assumptions, fitting model assumptions	The sead_7T_26ms_11Dec2013.BASIS' file supplied with MRspa was used. This basis set contains simulated spectra and a measured macromolecular baseline.

4. Data Quality and data loss	
a. Reported variables (SNR, Linewidth (with reference peaks))	Metabolite ratios relative to total creatine (creatine + phosphocreatine), SNR, FWHM, CRLB as reported in LCModel output file.
**b.** Data exclusion criteria and data loss	In order to avoid exclusion of values that are disorder/group specific and can provide insight into the nature of OCD, we followed Kreis (2016), and avoided using a straight cut-off score for CRLB. Instead, per metabolite and per group, the average and standard deviation were calculated for Cramér-Rao Lower Bound of each metabolite, and individual metabolite concentrations. Next, values larger than 2SD from each group's mean CRLB and concentration levels were excluded. The latter were according to Frangou et al. (2019). According to this criteria, the following data were excluded: within the SMA voxel, GABA in one healthy and one OCD subjects, and Gln in two OCD patients were excluded; within the OCC voxel, Glu and Gln in two healthy subjects, GABA in one healthy subject and three patients were excluded. One ACC and one OCC voxels were excluded for one OCD patient due to an error during data collection which led to loss of data. For one patient, the scan was stopped and the data was excluded for due to a panic attack. All spectra averages were visually checked by the first author MB and the noisy averages were removed. For the OCC voxel, in 3 patients, 1 average was removed and in 1 patient, 3 averages were removed out of 64. For the SMA voxel, in 2 OCD participants 2 and 3 averages were removed. Lastly, due to a mistake in data collection in one healthy volunteer 54 averages were collected instead of 64 for the ACC voxel.
**c.** Quality measures of postprocessing Model fitting	See above.
**d.** Sample Spectrum	Fig.4A in the main text shows sample spectra for GABA and Glutamate peaks.

List of p-values before FDR correction

a) Behavioral correlations between checking, accuracy and confidence (4 comparisons with the following p-values: 0.0008, 0.006, 0.10, 0.88)

b) Correlations between checking and clinical scales (8 comparisons: 0.005, 0.008, 0.01, 0.06, 0.07, 0.48, 0.58, 0.97)

c) Correlations between Glu/GABA levels and checking and accuracy in both groups, and per individual voxel (4 comparisons per voxel: ACC (0.007, 0.01, 0.36, 0.62), SMA (0.57, 0.61, 0.67, 0.73), OCC (0.22, 0.29, 0.34, 0.36)).

Table S3 Measures of magnetic resonance spectroscopy quality and tissue composition.

Measures	HV	OCD	t / *U*	DF	d	*p*	95% CI
	M ± SD	M ± SD					
**ACC**							
SNR	56.80 ± 6.09	60.97 ± 6.29	2.58	57	0.67	**0.01***	[0.94, 7.40]
FWHM	0.02 ± 0.00	0.020 ± 0.00	354 U	NA	0.033	0.17	[-0.005, 0.00]
GM	0.84 ± 0.02	.84 ± .04	0.007	57	0.002	0.99	[-0.017, 0.017]
WM	0.10 ± 0.03	0.10 ± 0.02	- 0.21	57	-0.055	0.83	[-0.014, 0.011]
CSF	0.05 ± 0.02	0.05 ± 0.03	380 U	NA	0.012	0.41	[-0.018, 0.008]
% CRLB GABA	9.17 ± 1.31	9.20 ± 1.18	407 U	NA	0.001	0.49	[0.00, 1.00]
% CRLB Glu	1.89 ± 0.31	1.83 ± 0.38	420 U	NA	0.008	0.45	[0.00, 0.00]
**SMA**							
SNR	57.20 ± 10.47	54.74 ± 13.51	- 0.78	58	-0.20	0.43	[-8.74, 3.81]
FWHM	0.02 ± 0.00	0.03 ± 0.01	472 U	NA	0.002	0.71	[0.00, 0.00]
GM	0.71 ± 0.06	0.07 ± 0.50	0.43	58	0.11	0.67	[-0.023, 0.036]
WM	0.17 ± 0.04	0.16 ± 0.04	- 0.78	58	-0.20	0.44	[-0.028, 0.012]
CSF	0.11 ± 0.06	0.11 ± 0.05	480 U	NA	0.003	0.65	[-0.019, 0.036]
% CRLB GABA	9.80 ± 2.91	9.55 ± 2.01	421 U	NA	0.000	0.99	[-1.00, 1.00]
% CRLB Glu	2.07 ± 0.25	2.19 ± 0.75	483 U	NA	0.002	0.68	[0.00, 0.00]
**OCC**							
SNR	80.79 ± 12.59	78.23 ± 18.79	- 0.61	58	-0.16	0.54	[-10.89, 5.75]
FWHM	0.03 ± 0.00	0.03 ± 0.00	466 U	NA	0.001	0.77	[0.00, 0.00]
GM	0.80 ± 0.03	0.81 ± 0.04	1.20	57	0.31	0.23	[-0.007, 0.030]
WM	0.16 ± 0.03	0.15 ± 0.03	- 1.05	57	-0.27	0.29	[-0.025, 0.008]
CSF	0.03 ± 0.01	0.03 ± 0.01	382 U	NA	0.011	0.42	[-0.019, 0.003]
% CRLB GABA	9.20 ± 2.14	10.53 ± 6.70	347 U	NA	0.000	0.60	[-1.00, 1.00]
% CRLB Glu	1.86 ± 0.44	1.79 ± 0.56	367 U	NA	0.006	0.62	[0.00, 0.00]

All the tests used were two-sides. The sample size for the ACC voxel for Glu and GABA was [OCD: n = 30, HV: n = 29]; for the SMA Glu it was [OCD: n = 31, HV: n = 29], and for GABA the sample size was [OCD: n = 30, HV: n = 28]; for OCC Glu it was [OCD: n = 30, HV: n = 27] and lastly, for OCC GABA the total number of participants were [OCD: n = 27, HV: n = 28]. Significant p-values are shown in bold. Acronyms: ACC = anterior cingulate cortex, SMA = supplementary motor area, OCC = occipital cortex, SNR: signal-to-noise ratio, FWHM = full width at half maximum in ppm units, GM = gray matter fraction, WM = white matter fraction, CSF = cerebrospinal fluid, % CRLB: percentage Cramer-Rao Lower Bound, GABA = γ-amino-butyric acid, Glu = glutamate, Gln = Glutamine, NAA = N-acetylaspartate, t = independent sample t-test, U = Mann-Whitney U test, M = mean, SD = standard deviation, NA = not applicable, $\eta p2$ = partial eta-square (a measure of effect size for the U test), DF = degree of freedom, d = Cohen's d, CI = Confidence Interval of the t-test, HV = healthy volunteers, OCD = obsessive-compulsive disorder, * = $p < 0.05$; .

Supplementary References

1. Lin, A., Andronesi, O., Bogner, W., Choi, I. Y., Coello, E., Cudalbu, C., ... & Mullins, P. G. (2021). Experts' Working Group on Reporting Standards for MR Spectroscopy. Minimum reporting standards for in vivo magnetic resonance spectroscopy (MRSinMRS): experts' consensus recommendations. *NMR Biomed*, *34*(5), e4484.
2. Biria, M., Banca, P., Healy, M. P., Keser, E., Sawiak, S. J., Rodgers, C. T., ... & Robbins, T. W. (2023). Cortical glutamate and GABA are related to compulsive behaviour in individuals with obsessive compulsive disorder and healthy controls. *Nature Communications*, 14(1), 3324.
